# Phytotoxic Interference of Culture Filtrates of Endophytic Bacteria Associated with *Nerium oleander* Leaf Against Seed Germination of the Invasive Noxious Weed *Cenchrus echinatus*

**DOI:** 10.1007/s00284-022-03166-z

**Published:** 2023-01-06

**Authors:** Noura Sh. A. Hagaggi, Usama M. Abdul-Raouf

**Affiliations:** Botany Department, Faculty of Science, Aswan University, Aswan, 81528 Egypt

## Abstract

Weeds cause destructive agricultural losses, so weed control is an urgent challenge facing agriculture. The extensive use of synthetic chemical herbicides has detrimental environmental impacts and promotes the emergence of resistant species. Therefore, in this study we tried to find a new natural weed control that can ensure biosafety and eco-sustainability. The phytotoxic potential of culture filtrates of the endophytes *Bacillus inaquosorum* NL1 and *Bacillus safensis* NL2 isolated from *Nerium oleander* leaf against the invasive harmful weed species *Cenchrus echinatus* was evaluated. Culture filtrates of both bacterial species exhibited potent phytotoxic activity, which resulted in 100% germination inhibition of *C. echinatus*. The chemical analysis of culture filtrates revealed high contents of total phenolics and n-alkanes that have phytotoxic effects against seed germination. According to the findings of this study the endophytic bacteria associated with *N. oleander* leaf can be used in the future to develop a sustainable bio-herbicide formulation.

## Introduction

The invasion of noxious weeds in agricultural lands causes serious problems that have negative impact on ecosystems, native gene pools and commercial crop productivity [1]. *Cenchrus echinatus* L. is known by the common name southern sandbur or spiny sandbur, is a notorious invasive and noxious weed which belongs to the family Poaceae. It is native distributed in Southern States of North America, Mexico, and South America, and it can easily invade a poorly managed fields in warm temperate, sub-tropical and tropical parts of the world diminishing the quality of crops and grazing lands [2]. The seeds of *C. echinatus* are easily dispersed by attaching their spines to animals, shoes, clothes, tires, or machinery [3].

Many chemical synthetic herbicides like metsulfuron, imazapic, glyphosate, fluazifop and indaziflam are used to control *C. echinatus* [4, 5]. The application of chemical herbicides causes environmental consequences such as soil, water, air, and food pollution [6]. Therefore, in the recent decades the herbicide discovery programs interest in alternative natural compounds with phytotoxic properties [7]. Although the use of plant extracts to control weeds is gaining attention in many parts of the world, it has the disadvantage that plants production is influenced by seasonal and environmental conditions [8]. Hence, microorganisms have been considered as a promising alternative source due to easy and fast controllable growth [9].

*Nerium oleander* (L.) is a worldwide cultivated toxic shrub that belongs to the family Apocynaceae. It was selected for this study due to its allelopathic properties because it produces several phytotoxic metabolic compounds [10]. Since endophytes can produce metabolic compounds in the same way as their host plants [11], we expected that the endophytic bacteria associated with *N. oleander* may produce phytotoxic compounds like host plant; therefore, the hypothesis of the current study is that endophytic bacteria isolated from leaves of *N. oleander* would be promising source of phytotoxic agents that could be effectively used for controlling weeds.

In the last years and with the expansion of greenhouses construction in our university, an annoying problem has emerged, which is the infestation of noxious weed *Cenchrus echinatus*. Its spiny seeds can attach to clothes and shoes of students and penetrate the skin causing painful injuries. This study was aimed to isolate the endophytic bacteria associated with *N. oleander* leaf and to investigate the phytotoxic effect of their culture filtrates on the seed germination of noxious weed *C. echinatus* with a future aim to exploit these bacteria for developing a new natural bio-herbicides.

## Materials and Methods

### Plant Material

Healthy leaves of Oleander (*Nerium oleander* L.) were collected from Aswan University campus, Egypt (24° 5′ 26.95″ N, 32° 53′ 57.91″ E), and were immediately transferred to laboratory for bacterial isolation.

### Isolation of Endophytic Bacteria

The surface sterilization of leaves was performed using sodium hypochlorite (5%) for 1 min and ethanol (70%) for 1 min and then washed three times with sterilized distilled water; one gram of leaf tissue was crushed in 9 mL sterilized saline solution; one milliliter of suspension was spread into tryptic soy agar and nutrient agar plates; one milliliter of the sterilized distilled water resulted from the final rinse during surface sterilization process was also spread on the same media to confirm sterilization success. Plates were incubated at 37 °C for 72 h until the appearance of colonies. The obtained isolates were coded as NL1 and NL2.

### Confirmation of Endophytic Association Between *N. oleander* and the Isolated Strains

The endophytic association between *N. oleander* and the isolated strains was confirmed by aseptically re-inoculation of the isolated strains in healthy seedlings following the method of Gagne-Bourgue et al. [12]. Three-week-old *N. oleander* seedlings were obtained from agriculture department, Aswan University. The soil was obtained from Arid Lands Plants Study Unit, Aswan University. Soil texture was clay: sand in a ratio of 2:1. pH, organic matter content and moisture content of the soil were 6.8, 4.2% and 41% respectively. Five seedlings were transplanted in pots filled with autoclaved soil. Pots were assigned to three groups, the first group which received 200 mL water containing 10^7^ CFU/mL of strain NL1, the second group received 200 mL water containing 10^7^ CFU/mL of strain NL2 and the third group that received 200 mL autoclaved distilled water served as control. Inoculum was applied into the soil surrounding plant roots. Pots were kept under normal weather conditions at May 2022 (Temperature, 38 ± 2 °C, humidity, 23%, pressure, 1008 hPa and wind speed, 5 km/h). Five replicate pots were made for each treatment. After 4 weeks of inoculation, the strains were re-isolated from leaves tissue under controlled conditions and compared with the strains isolated from the original samples.

### Identification of Endophytic Bacteria

The partial sequencing of 16S rRNA gene was used to identify the isolates at the molecular level. Using the bacterial primers 27F and 1492R, the 16S rRNA gene was amplified by PCR [13]. Products of PCR were separated by agarose gel electrophoresis. The obtained bands were sequenced in Korea Solgent Company. The percentage of sequence identity was determined using BLAST tool in NCBI. Sequences were recorded in NCBI with accession numbers. Phylogenetic analysis was carried out by MEGA X software [14].

### Preparation of Culture Filtrates

One milliliter of bacterial suspension (⁓10^7^ CFU/mL) was inoculated into 200 mL of nutrient broth and incubated for 72 h at 37 °C with shaking (150 rpm). Cultures were centrifuged and the supernatants were filtered twice using 0.22 µm membrane filter. The obtained filtrates were stored at 4 °C for further study.

### Collection of Weed Seeds

The seeds of *C. echinatus* were collected from different localities in Aswan region, Egypt. Seeds were surface sterilized with 5% sodium hypochlorite (1 min), rinsed three times by sterilized distilled water and aseptically dried in the laboratory at room temperature [15].

### Seed Germination Bioassay

Seeds of *C. echinatus* were subjected to three treatments which included culture filtrate of isolate NL1, culture filtrate of isolate NL2 and distilled water as control. For each treatment thirty seeds were placed in 12 cm Petri dishes contained autoclaved filter paper wetted with 10 mL of the tested filtrate or 10 mL distilled water (control). Plates were incubated at 28 °C and daily observed for 10 days. Radicle emergence was considered as germination. The percentage of germination inhibition (GI) was calculated according to Ranal et al. [16] as follows:

$$\mathrm{GI }\left(\mathrm{\%}\right)=\frac{\mathrm{Gc}-\mathrm{Gt}}{\mathrm{Gc}} \times 100$$

where Gc is the average number of seeds germinated in control; and Gt is the average number of seeds germinated in treatment. The assay was repeated three times under the same conditions with five replicates per treatment.

### Estimation of Enzymatic Activity in Treated Seeds

#### Extraction of Enzymes

The method of Elarbi et al. [17] was followed to extract enzymes from treated seeds. One gram (about ten seeds) of 4 days old- germinated seeds from each treatment group were homogenized in 10 mL of 0.1 M acetate buffer (pH 6) and then stirred slowly for 1 h at 4 °C. The homogenates were centrifuged for 15 min at 5000 rpm at 4 °C. The obtained supernatants (crude enzymes) were stored at 4 °C in the refrigerator for further assays.

#### Estimation of Amylase

Amylase activity was estimated using the iodometric method described by Xiao et al. [18]. In this method, 1 mL of crude enzyme was added to 1 mL of 0.2% soluble starch solution dissolved in 0.01 M sodium phosphate buffer pH 6.9. The reaction was incubated for 30 min at 37 °C. After that, the reaction was stopped using 1 mL of 0.6 N trichloroacetic acid (TCA). The amylase activity was measured spectrophotometrically at 580 nm. The activity was calculated in IU/g seeds using amylase (Blulux™) standard curve. One unit of activity was defined as the amount of enzyme that hydrolyses 0.2 g of starch to dextrin per minute under the reaction conditions.

#### Estimation of Protease

The activity of protease in seeds was assessed according to the method of Mushtaq et al. [19]. One milliliter of 0.5% (w/v) casein prepared in 0.1 M Tris–HCl buffer (pH 8.0) was incubated with 1 mL of the crude enzyme at 37 °C for 30 min. Then the reaction was stopped using 5 mL of 10% (w/v) trichloroacetic acid (TCA). The reaction mixture was centrifuged for 15 min at 5000 rpm and 4 °C. To 1 mL of the collected supernatant, 2.5 mL of 0.4 M Na_2_CO_3_ and 0.5 mL of 1 N Folin–Ciocâlteu reagent were added, mixed by swirling, and incubated at 37 °C for 30 min. The absorbance was measured at 660 nm and L-tyrosine was used as standard. One unit of enzyme activity was defined as the amount of enzyme required to release 1 μmol of tyrosine equivalents from casein per minute under standard assay conditions.

#### Estimation of Lipase

To evaluate the activity of lipase in treated seeds, the reaction mixture containing 1 mL of crude enzyme, 8 mL of 50 mM Tris–HCl buffer (pH 8), and 1 mL of 10 mM p-nitrophenyl-palmitate (pNPP) was incubated for 1 h at 37 °C. The reaction was stopped using 1 mL of 0.6 N TCA. Absorbance was read at 410 nm [20]. Lipase standard curve was used to evaluate enzyme activity as IU/g seeds. One unit of enzyme activity was defined as the amount of enzyme that released 1 μmol of p-nitrophenol equivalents from pNPP per minute under standard assay conditions.

### Estimation of Phytohormones in Treated Seeds

#### Extraction of Phytohormones

Upon fourth day of germination, 1 g of seeds from each treatment group was frozen and then homogenized. The homogenates were extracted with 5 mL of cold 80% (v/v) methanol, thoroughly vortexed and left at 4 °C overnight [21]. Solids were removed by centrifugation (5000 rpm, 20 min, 4 °C) and re-extracted with 5 ml of the same solvent. Supernatants were collected and used for phytohormones evaluation.

#### Estimation of Gibberellic Acid (GA3)

Gibberellic acid was estimated using the method of Berríos et al. [22]. Briefly, in 25 mL- flasks 1 mL of the sample was added to 1 mL of absolute ethanol and the volume was completed to 10 mL with 3.75 M HCl and vigorously mixed for 10 s. The absorbance was measured at 254 nm. The amount of GA3 in the samples was calculated using GA3 standard curve.

#### Estimation of Indole Acetic Acid (IAA)

IAA was assayed by Salkowsky’s colorimetric method [23]. One milliliter of the sample was mixed with 1 mL Salkowski reagent, vortexed gently and incubated for 30 min in a dark condition. Absorbance was measured at 530 nm. A curve of standard IAA was used to calculate the concentration of IAA in the samples.

### Chemical Composition of Culture Filtrates

#### Estimation of Total Phenolic Content

Folin-Ciocalteu method [24] was used to evaluate the total phenolic content in the culture filtrates of isolate NL1 and NL2. Briefly, 1 mL of the culture filtrate was mixed with 2 mL of Folin-Ciocalteu reagent and 1 mL of 10% sodium carbonate solution. The mixture was incubated for 1 h with shaking at room temperature until the appearance of blue color. Absorbance was measured at 700 nm. The content of total phenolic was calculated as mg gallic acid equivalent/mL filtrate using gallic acid standard curve.

### GC–MS Analysis

The chemical profile of the tested culture filtrates was determined using TRACE™ TR-5MS GC column (Thermo Scientific, ICE3000). Samples were carried by Helium at 1.0 mL per minute flow rate. The starting temperature of the column was 60 °C for 1 min, and then raised up to 240 °C at a rate of 4.0 °C/min. Hexane was used to dilute the samples at the ratio of 1:10 (v/v). One microliter of the diluted samples was injected. The unknown compounds in the samples were identified by matching their mass spectra and retention indices with those of a standard mixture of C7-C40 n-alkanes and NIST standards.

### Statistical Analysis

The obtained results were analyzed by one-way analysis of variance (ANOVA) using the Minitab 18 software. The significant differences between the control and the treatments were considered at *P* ≤ 0.05. Correlation coefficient (*r*) between enzymes activities, phytohormones contents and germination inhibition (GI) was calculated using data analysis tool in Excel, and the obtained results were visualized using heat mapper web server [25].

The 16S rRNA gene sequences of the isolate NL1 and NL2 were deposited in the NCBI database with accession numbers ON714987.1 and ON714988.1, respectively.

## Results

### Identification of Endophytic Bacteria

**Fig. 1:**
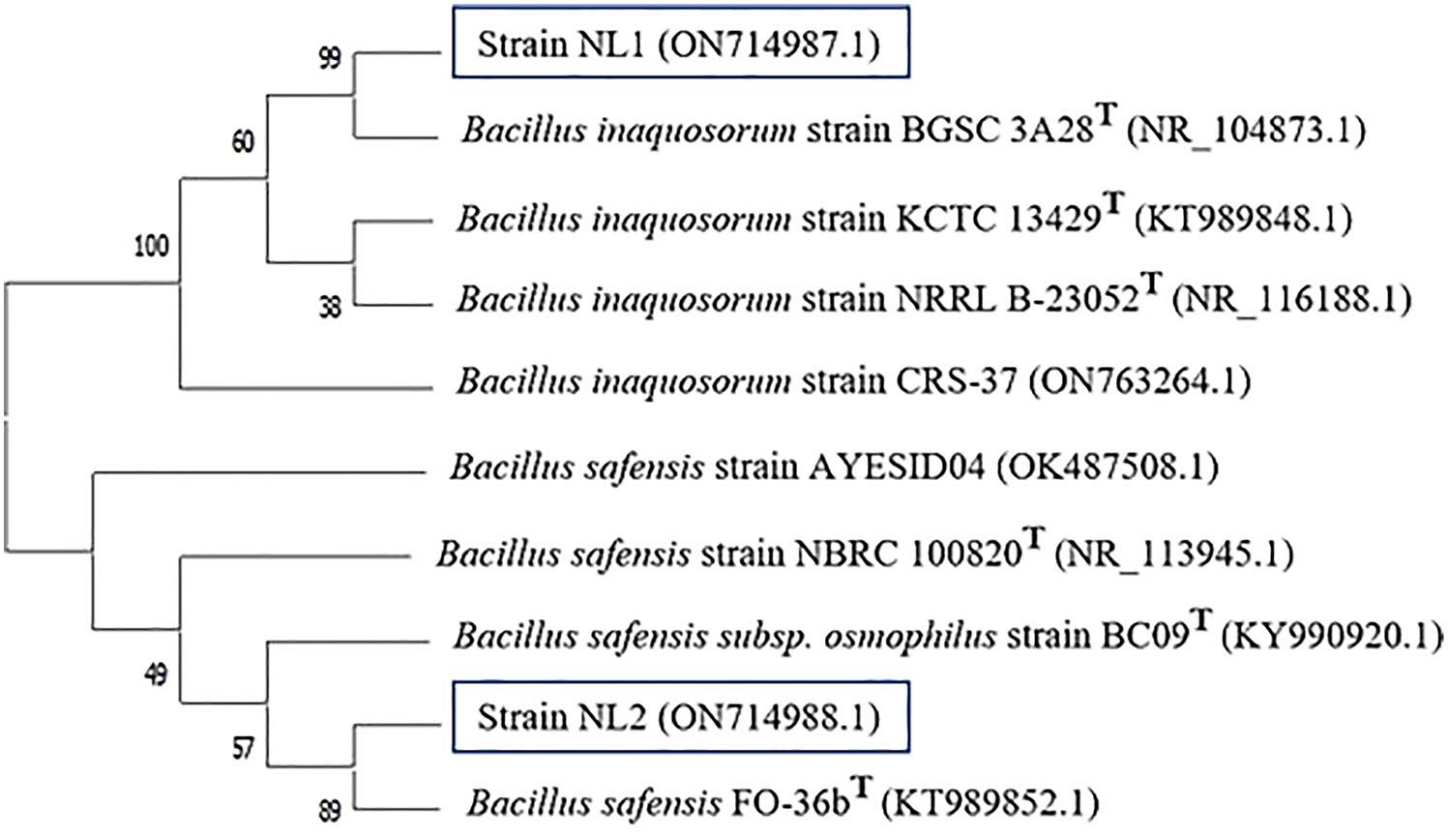
Phylogenetic relationships between strain NL1, strain NL2 and the most related members of genus *Bacillus* from NCBI database

Based on the NCBI-BLAST analysis, the 16S rRNA sequences of the endophytic bacterial isolates NL1 and NL2 showed close similarity to *Bacillus inaquosorum* strain BGSC 3A28^T^ (NR_104873.1) and *Bacillus safensis* FO-36b^T^ (KT989852.1), respectively. Neighbor-joining phylogenetic tree (Fig. 1) was inferred using the sequences of *Bacillus* species derived from NCBI GenBank that were aligned with the sequences of the present isolates.

### Effect of Culture Filtrates on Seed Germination of *C. echinatus*

**Fig. 2:**
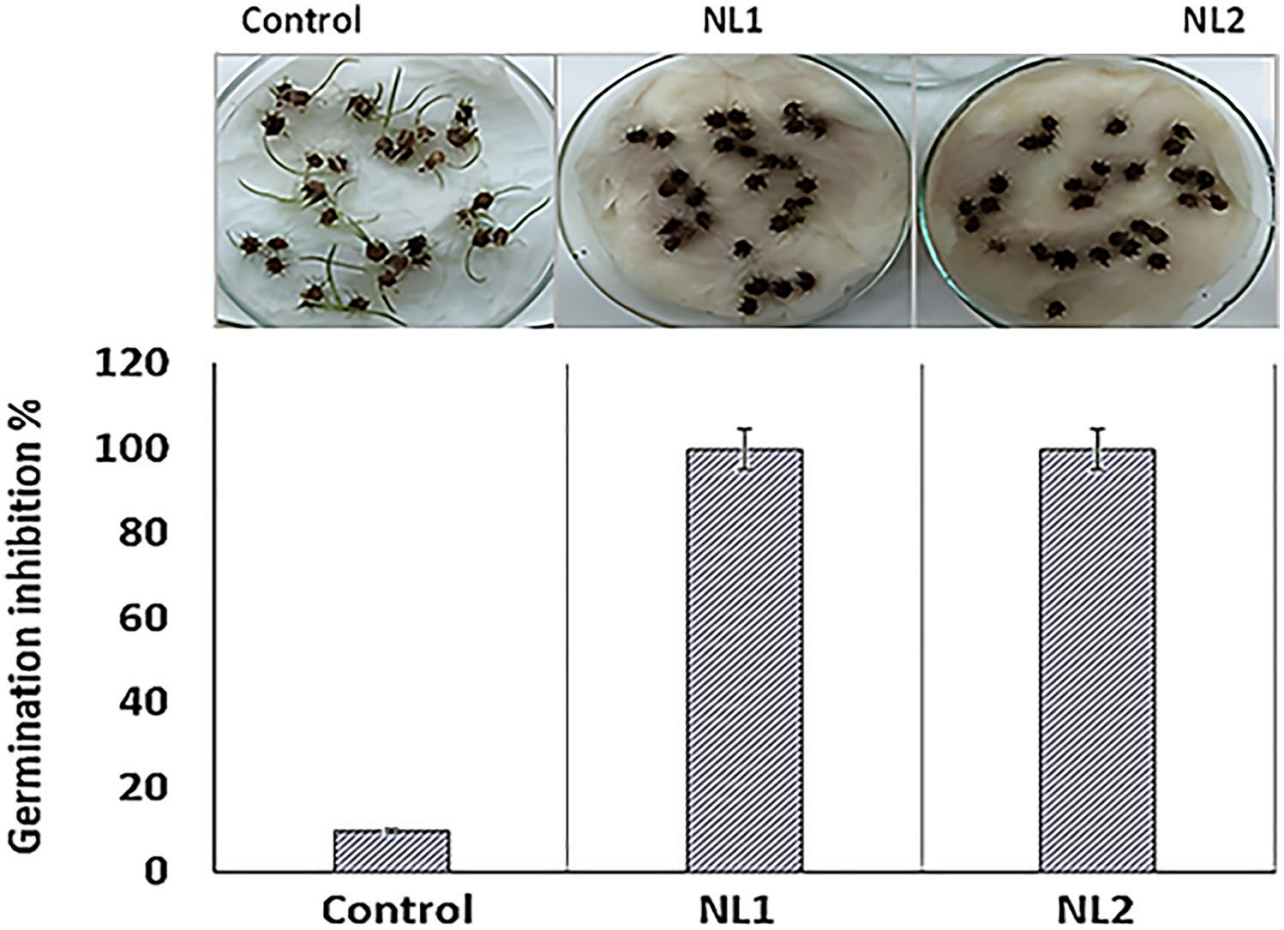
The effect of culture filtrates of *B. inaquosorum* NL1 and *B. safensis* NL2 on germination inhibition of *C. echinatus* seeds. Filtrates of both NL1 and NL2 completely inhibited (100%) germination compared to the control. Standard errors of the means of five biological replicates (*n* = 5) are shown for each graph bar as vertical lines

The effect of culture filtrates of endophytic isolate NL1 and NL2 on seed germination of *C. echinatus* was assessed. Both culture filtrates showed significant (*f* = 1849, *P* < 0.00001) inhibitory effect against seed germination, where germination was completely inhibited (GI = 100%) upon the treatment with the bacterial filtrates (Fig. 2).

### Effect of Culture Filtrates on Enzymatic Activities and Phytohormones Content of the Seeds

**Fig. 3:**
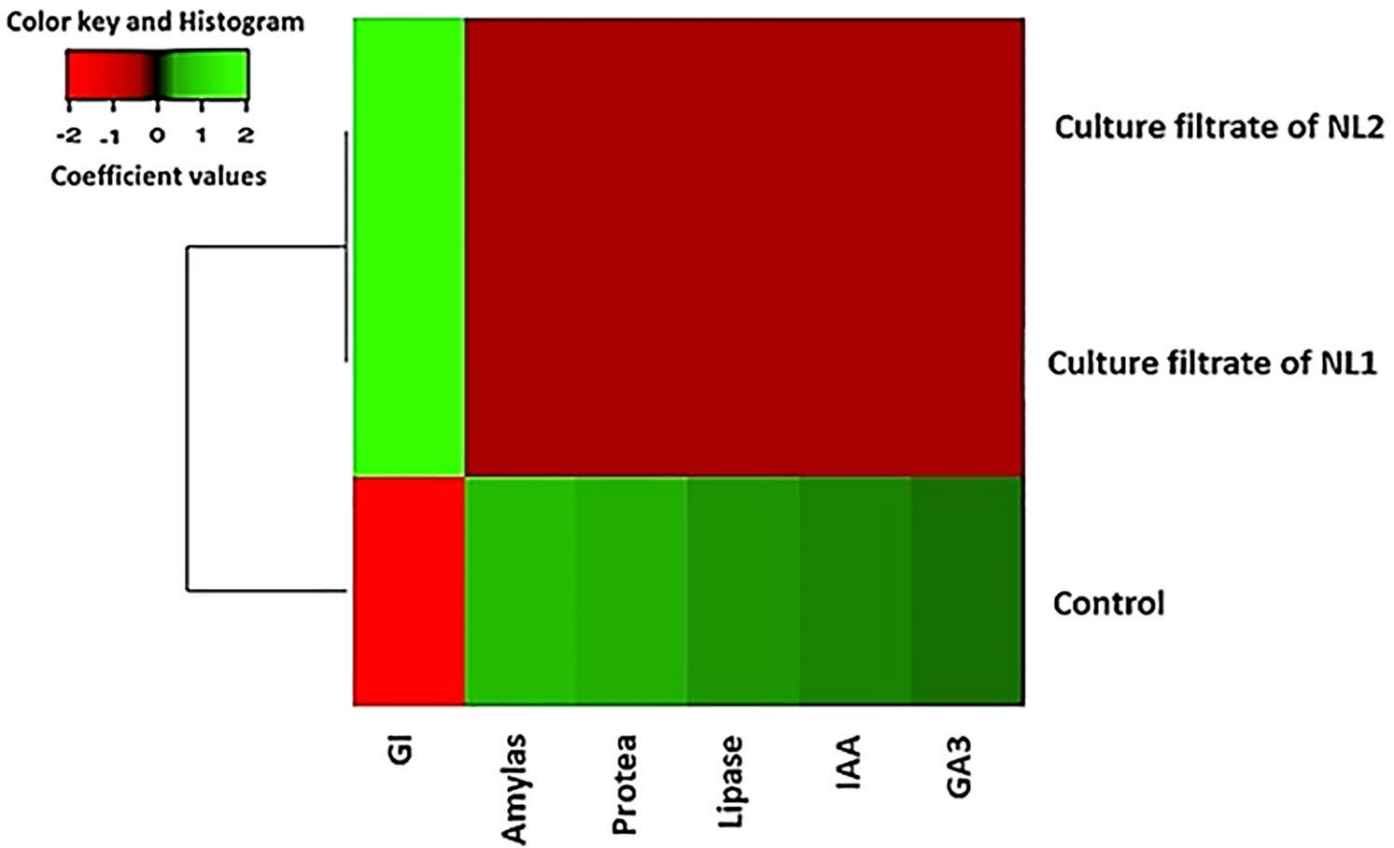
Heatmap illustrates the inverse correlation between enzymes activities, phytohormones contents and germination inhibition (GI) (When enzymes activities and phytohormones contents are high, the germination inhibition is low and vice versa). The green color indicating high values and red color indicating low values. In control group, the enzymes activities as well as phytohormones contents were high (green color), so GI was low (red color), while in seeds treated with filtrates of both NL1 and NL2, the enzymes activities and phytohormones were suppressed (red color), so GI was high (green color) (Color figure online)

The activities of amylase, protease, and lipase as well as the contents of IAA and GA3 of the treated seeds and their respective controls were estimated. Interestingly, treatment of seeds with culture filtrates had significant positive effect on enzymes (*f* = 18.16056, *P* = 0.000099) as well as phytohormones (*f* = 360.16108, *P* < 0.00001), where enzymes activities and phytohormones production were completely suppressed by culture filtrates which resulted in germination inhibition. There was an inverse correlation between enzymes activities, phytohormones content and germination inhibition (Fig. 3). When enzymes activities and phytohormones content are high, the germination inhibition is low and vice versa.

### Chemical Composition of Culture Filtrates

High total phenolic contents were estimated in the culture filtrates of isolate NL1 and NL2 as 39.3 ± 0.01 and 36.84 ± 0.04 mg gallic acid equivalent /mL filtrate respectively.

After matching the mass spectra and retention indices of the compounds detected in the culture filtrates against NIST library, it was found that the major compounds identified in culture filtrate of *B. inaquosorum* NL1 were Heptane, 2,2,4,6,6-pentamethyl-, Decane, 3-methyl-, Eicosane, Octadecane, Tridecane, 3-methyl-, Octadecane, 2-methyl-, Hexadecane, Heptadecane, Heneicosane, Hentriacontane, Octacosane, 7,9-Di-tert-butyl-1-oxaspiro (4,5) deca-6,9-diene-2,8-dione and 3,5-Dimethyldodecane (Table 1). The main components that were identified in the culture filtrate of *B. safensis* NL2 being Heptane, 2,2,4,6,6-pentamethyl-, Decane, 2,4,6-trimethyl-, Sulfurous acid, 2-propyl tetradecyl ester, Heptadecane, 2,6,10,15-tetramethyl-, Tetradecane, Eicosane, Decane, 3,8-dimethyl-, Tridecane, 1-iodo-, Octadecane, Pentacosane, Undecane, Benzoic acid, 2,5-bis (trimethylsiloxy)-, trimethylsilyl ester, Heptacosane, Tetratetracontane, Heptadecane, 2-methyl-, 7,9-Di-tert-butyl-1-oxaspiro (4,5) deca-6,9-diene-2,8-dione, Dibutyl phthalate (Table 2).

**Table 1:** Chemical composition of culture filtrate of *B. inaquosorum* NL1

Compound name	Formula	Molecular weight	Retention time	Peak area %
Heptane, 2,2,4,6,6-pentamethyl-	C_12_H_26_	170	4.430	3.089
Decane, 3-methyl-	C_11_H_24_	156	7.378	3.158
Eicosane	C_20_H_42_	282	9.089	11.009
Octadecane	C_18_H_38_	254	9.169	2.002
Tridecane, 3-methyl-	C_14_H_30_	198	9.495	10.374
Octadecane, 2-methyl-	C_19_H_40_	268	9.593	2.854
Hexadecane	C_16_H_34_	226	10.062	2.582
Heptadecane	C_17_H_36_	240	11.200	2.199
Heneicosane	C_21_H_44_	296	11.378	13.224
Hentriacontane	C_31_H_64_	436	12.064	2.348
Octacosane	C_28_H_58_	394	14.531	7.123
7,9-Di-tert-butyl-1-oxaspiro (4,5) deca-6,9-diene-2,8-dione	C_17_H_24_O_3_	276	14.571	5.104
3,5-Dimethyldodecane	C_14_H_30_	198	15.240	7.510

**Table 2:** Chemical composition of culture filtrate of *B. safensis* NL2

Compound name	Formula	Molecular weight	Retention time	Peak area %
Heptane, 2,2,4,6,6-pentamethyl-	C_12_H_26_	170	4.431	5.271
Decane, 2,4,6-trimethyl-	C_12_H_26_	184	5.215	2.061
Sulfurous acid, 2-propyl tetradecyl ester	C_17_H_36_O_3_S	320	7.378	4.788
Heptadecane, 2,6,10,15-tetramethyl-	C_21_H_44_	296	7.733	4.154
Tetradecane	C_14_H_30_	198	7.807	1.872
Eicosane	C_20_H_42_	282	9.089	14.139
Decane, 3,8-dimethyl-	C_12_H_26_	170	9.169	2.768
Tridecane, 1-iodo-	C_13_H_27_I	310	9.495	12.397
Octadecane	C_18_H_38_	254	9.593	3.417
Pentacosane	C_25_H_52_	352	9.684	2.545
Undecane	C_11_H_24_	156	10.062	2.818
Benzoic acid, 2,5-bis(trimethylsiloxy)-, trimethylsilyl ester	C_16_H_30_O_4_Si_3_	370	10.789	2.007
Heptacosane	C_27_H_56_	380	11.378	10.455
Tetratetracontane	C_44_H_90_	618	11.939	8.578
Heptadecane, 2-methyl-	C_18_H_38_	254	14.531	7.405
7,9-Di-tert-butyl-1-oxaspiro (4,5) deca-6,9-diene-2,8-dione	C_17_H_24_O_3_	276	14.571	3.501
Dibutyl phthalate	C_16_H_22_O_4_	278	15.240	9.824

## Discussion

Weed infestation is among the main factors contribute to the severe losses of annual agricultural productivity [26]. Due to the serious negative impacts of conventional weed control using traditional chemical herbicides on the environment, human health, soil, and groundwater, the interest in alternative natural compounds with phytotoxic properties for safer weed management practices has grown in recent decades [27]. The use of bacteria for controlling weeds has been suggested to have several advantages over the use of plant extracts and fungi due to their rapid growth, relatively simple propagation requirements and high suitability for genetic modification [28, 29].

In this study we tried to find new natural promising solution to control the invasive noxious weed species *C. echinatus* that can ensure biosafety and eco-sustainability. Two endophytic bacterial strains *B. inaquosorum* NL1 and *B. safensis* NL2 were isolated from *N. oleander* leaf. It was observed that *N. oleander* leaf colonized by a few numbers of endophytic bacteria, this may be due to its antimicrobial activity [30]. This was also observed in other medicinal plants which possess antimicrobial activity [31]. The two isolated strains NL1 and NL2 were related to genus *Bacillus*. It was previously reported that genus *Bacillus* is one of the most prevalent genera of endophytic bacteria associated with medicinal plants [32]. This may be attributed to that members of genus *Bacillus* are characterized by their ability to form heat-resistant endospores, which can survive in the extremely hot climate of Aswan region and can also resist the antimicrobial activity of *N. oleander* leaf.

In the present study the effect of culture filtrates of the endophytic bacteria *B. inaquosorum* NL1 and *B. safensis* NL2 on the germination of *C. echinatus* seeds was investigated. The results revealed that the culture filtrates of both bacterial strains completely inhibited germination of *C. echinatus* seeds compared to the control (Fig. 2). This agreed with the findings of the other workers who reported that culture filtrates of *Pseudomonas fluorescens* strain D7 and WH6 that were isolated from winter wheat (*Triticum aestivum*) and downy brome (*Bromus tectorum*) have been recognized to inhibit germination and growth of several grassy weeds [33, 34]. It was previously reported that the endophytic *Xanthomonas campestris* pv. poae was used to control the annual bluegrass (*Poa annua*) in Japan [35]. Furthermore, *X. campestris* LVA-987 was recognized as a potential control agent against horseweed (*Conyza canadensis*) [36].

Nutrient mobilization process in the seed that supplies energy and other fundamental food materials to germinating embryo through solubilizing the stored starch, proteins, and lipids is achieved by enzymes such as amylase, protease, and lipase [37]. On the other hand, phytohormones such as gibberellic acid and indole acetic acid can stimulate seed germination through releasing coat dormancy, weakening endosperm, and expanding embryo cell [38]. In the current study, the activities of amylase, protease, and lipase as well as the contents of IAA and GA3 in the treated seeds and their respective controls were estimated as an attempt to find a possible mechanism explaining the reasons of germination inhibition caused by bacterial filtrates. Pearson’s correlation analysis of the obtained results showed that the germination inhibition occurred due to the complete suppression of enzymes and phytohormones upon the treatments with bacterial filtrates (Fig. 3). Suppression of enzymes and phytohormones in the treated seeds may be related to the high contents of total phenolics detected in the seeds, where other researchers reported that phenolic compounds influence the physicochemical properties and consequently the activity of enzymes, as well as cause variations in the hormonal balance [39, 40]. Furthermore, the GC–MS analysis of the culture filtrates of both *B. inaquosorum* NL1 and *B. safensis* NL2 revealed that the filtrates are rich in n-alkanes (saturated hydrocarbons) as shown in Tables 1 and 2. Phytotoxicity of alkanes against seed germination and plants development is well known [41–43].

## Conclusion

Culture filtrates of the endophytes *B. inaquosorum* NL1 and *B. safensis* NL2 isolated from *N. oleander* leaf have distinctive phytotoxic potential against the invasive harmful weed species *C. echinatus*. The findings obtained from this study suggest the high possibility of developing eco-friendly bio-herbicides from the culture filtrates of these bacteria. However, further studies are necessary to evaluate the possible application under field conditions.

## Data Availability

Not applicable.
